# Supplementary material for: Ptychography retrieval of fully polarized holograms from geometric-phase metasurfaces
Source: Nat Commun. 2020 May 27;11:2651. doi: 10.1038/s41467-020-16437-9 (PMC7253437; doi:10.1038/s41467-020-16437-9)
Supplement: Supplementary file 1 — Supplementary Information [file 41467_2020_16437_MOESM1_ESM.docx]

Supplementary Information

Ptychography Retrieval of Full-Polarized Holograms from Geometric-Phase Metasurfaces

Qinghua Song^1^, Arthur Baroni^2^, Rajath Sawant^1^, Peinan Ni^1^, Virginie Brandli^1^, Sébastien Chenot^1^, Stéphane Vézian^1^, Benjamin Damilano^1^, Philippe de Mierry^1^, Samira Khadir^1^, Patrick Ferrand^2^, and Patrice Genevet^1†^

*^1^ Université Cote d’Azur, CNRS, CRHEA, Rue Bernard Gregory, Sophia Antipolis 06560 Valbonne, France*

*^2^ Aix Marseille univ, CNRS, Centrale Marseille, Institut Fresnel, 13013 Marseille, France*

*^†^ Corresponding Author: Patrice.Genevet@crhea.cnrs.fr*

**Supplementary Figure 1. Phase-only superposition of LCP and RCP beams.** (a) Two supercells corresponding to LCP and RCP with different starting orientation angle that introduce a phase difference of 2*δ* between the two CP beams, generating a SoP of $|\left. n \right\rangle=|\left. R \right\rangle+e^{-i2\delta}|\left. L \right\rangle$. The obtained $|\left. n \right\rangle$ can be decomposed to two orthogonal LP as $E_{x}=\frac{1}{\sqrt{2}}\left( e^{-i2\delta}+1 \right)$ and $E_{y}=\frac{i}{\sqrt{2}}\left( e^{-i2\delta}-1 \right)$. The azimuth angle *ψ* and ellipticity angle *χ* are obtained as $\tan\left( 2\psi\right)=\frac{2E_{0x}E_{0y}}{E_{0x}^{2}-E_{0y}^{2}}\cos\left( \delta_{x}-\delta_{y} \right)=\tan(2\delta)$, and $\sin\left( 2\chi\right)=\frac{2E_{0x}E_{0y}}{E_{0x}^{2}+E_{0y}^{2}}\sin\left( \delta_{x}-\delta_{y} \right)=0$, where *E*_0_*_x_* and *E*_0_*_y_* are the amplitude of *E_x_* and *E*_y_, respectively; $\delta_{x}$ and $\delta_{y}$ are the phase of *E_x_* and *E_y_*, respectively. It is shown that the ellipticity angle *χ* is always equal to 0, so that no EP can be generated. (b) Plotted Poincaré sphere of the SoP when *δ* is changed from -π to π, indicating that only LP can be obtained by using phase-only superposition approach. Since the dimension of the building blocks are uniform for phase-only superposition method, the metasurface is broadband for all of the output linear polarizations. It becomes narrow band when the dimension of the building block in the bottom and top lines are not the same.


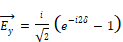

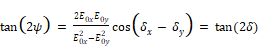

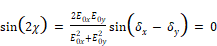

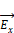

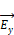

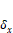

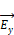

**Supplementary Figure 2. Phase encoding into pixelated deflector.** (a) – (d) The deflector is pixelated with *n* × *n* unit-cells with an orientation angle increment of *φ_d_*, which deflects the beam to the direction of $\theta_{t}=\arcsin\left( \frac{2\varphi_{d}}{k_{0}p} \right)$ according to Eq. 2. The starting orientation angle (SOA) of the pixelated deflectors varied pixel by pixel and the rest orientation angle in the corresponded sub-pixel is determined from Eqs. 1 and 2 according to the SoP and deflected angle. (e) When the adjacent sub-pixels are placed with the SOA relationship of (*φ*_2_ – *φ*_1_) = *m*π (*m* = ±1, ±2 …), no phase difference between the sub-pixels is introduced and (f) the wavefront of the adjacent sub-pixels are continuous. (g) When the adjacent sub-pixels are placed with the SOA relationship of (*φ*_2_ – *φ*_1_) ≠ *m*π, a phase difference of ± 2(*φ*_2_ – *φ*_1_) ≠ ±2*m*π is introduced and (h) the wavefront from the adjacent sub-pixels are no longer continuous that can be used to encode the holographic phase profile.


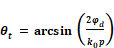

**Supplementary Figure 3. Pixelated deflector array for meta-hologram.** The meta-hologram with pixelated deflector is realized by patterning the sub-pixels into an array with different SOA of *φ_i_*_,_*_j_*, which contains both information of the deflector and holographic phase profile that follows the equation of $\varphi_{i,j}=-n(j-1)\varphi_{d}\mp\varphi_{i,j\left( holo \right)}/2$. The first term $-n(j-1)\varphi_{d}$ is the phase profile for conventional beam deflector and the second term $\varphi_{i,j\left( holo \right)}/2$ $\varphi_{i,j\left( \mathrm{holo} \right)}/2$ is for holographic phase profile. $\mp$ is due to opposite PB phase response of the two CP. If the bottom supercell is designed for LCP, we take negative sign. Otherwise, it is positive.


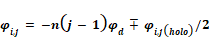

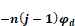

**Supplementary Figure 4. Determination of the number of meta-molecules within one supercell.** (a) Assume that there are *N* point light sources with phase increment of -*φ*, the electric field scattered from each source is $e^{-ij\varphi}$ (*j* = 1, 2…*N*). Therefore, the far field pattern $\vec{E_{f}}$ can be described as the sum of all sources, i.e., $\vec{E_{f}}=\sum_{j=1}^{N} e^{-ij\varphi}e^{ik(\left| \vec{r_{j}} \right|-\left| \vec{r} \right|)}=\sum_{j=1}^{N} e^{-ij\varphi}e^{ik\left( \frac{N+1}{2}-j \right)p\sin\omega}$, where *k* is the wavenumber in free space, *p* is the period of the array,$\left| \vec{r} \right|$ is the distance between the origin and the far field in the direction angle of $\omega$, $\left| \vec{r_{j}} \right|$ is the distance between the *j*th source and the far field in the direction angle of $\omega$. A normalized electric field pattern $\vec{E_{fn}}$ by dividing $\vec{E_{f}}$ by *N* is used to compare the intensity profile with different *N*. Considering the working wavelength of $\lambda=600 nm$, period of $p=300 nm$, wavenumber of $k=2\pi/\lambda$, the normalized far field pattern $\vec{E_{fn}}$ can be described as $\vec{E_{fn}}=\frac{\sum_{j=1}^{N} e^{-ij\varphi}e^{i\pi\left( \frac{N+1}{2}-j \right)\sin\omega}}{N}, N=2, 3, 4\ldots$ (b) The normalized far field pattern with different numbers of sources *N*. The phase differences between each source is chosen as $\varphi=60^{\circ}$. It is shown that when *N* is changed from 2 to 6 (i.e., the phase difference within a supercell is changed from 120° to 360°), the radiation angle always keeps at 19.5°, while the intensity radiated to the interested angle is enhanced. However, when *N* becomes larger, the size of the pixel is increasing, which decreases the total number of the pixels. As a trade-off, we choose *N* = 4 to design the pixels.

**Supplementary Figure 5. Simulation results of the GaN nano-pillars on sapphire substrate.** (a) The top view of a meta-molecule. (b) The transmission phase difference between *φ_x_* and *φ_y_* when the metasurface is impinged by *x*- and *y*-polarized light, respectively. (c) The transmission of *x*-polarized light |*E_x_*|. (d) The transmission of *y*-polarized light |*E_y_*|. The star shown in (b-d) represents the selected size for the structure in this paper with *L_x_* = 230 nm and *L_y_* = 120 nm, where |*E_x_*| and |*E_y_*| are near-unity and (*φ_x_* ̶ *φ_y_*) is π, so that the structure acts as a perfect half waveplate to convert the circular polarized light.

**Supplementary Figure 6. Simulated results of the magnetic field distribution.** (a) *H_x_* and (b) *H_y_* with the illumination of *y*- and *x*-polarized light, respectively. (c) *H_x_* and *H_y_* distribution along *z*-direction at *y* = 0. It can be observed that there are 5 and 5.5 oscillations for *H_x_* and *H_y_* in GaN nano-pillars, respectively, leading to a π phase difference between *H_x_* and *H_y_*. (d) *H_y_* distribution along *x*-direction at *z* = 185 nm. It is seen that the magnetic field is strongly confined in the GaN nano-pillars, resulting in a relatively weak coupling between adjacent meta-molecules.

**Supplementary Figure 7. Simulated results of the polarization conversion efficiency from LCP to RCP when the geometric size of GaN nano-pillars is chosen as *L_x_* = 230 nm and *L_y_* = 120 nm,** (a) when the wavelength of the incident light is changed from 450 nm to 850 nm with *p* = 300 nm. (b) when the period is changed from 250 nm to 450 nm with incident wavelength of *λ* = 600 nm. The conversion efficiency is higher than 90% when the period is between 275 nm and 340 nm. It drops fast when the period is larger than 350 nm due to diffraction in the substrate. We choose *p* = 300 nm in this paper.

**
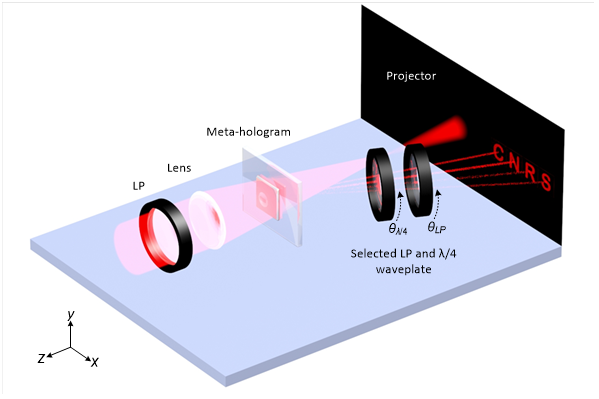
**

**Supplementary Figure 8. Optical setup for the measurement of meta-hologram.** A laser beam with diameter of 3 mm at a wavelength of 600 nm propagates through a linear polarizer and lens to weakly focus on the meta-hologram. The holographic images are projected onto the projector. Selected linear polarizer and quarter waveplate are placed before the images to analyze the SoP of selected letters.

**Supplementary Figure 9. Vectorial ptychography.** (a) The meta-hologram is scanned under a coherent spatially limited illumination. Series of intensity diffraction patterns are recorded by a camera as a function of the sample shift, under different polarization and analysis configurations. (b) The iterative vectorial ptychographic algorithm reconstructs the Jones matrix maps of the meta-hologram. (c) Jones matrix map are further used to compute the spatial distribution of the exit-field of the meta-hologram, and to perform numerically a far-field propagation, yielding to an exhaustive knowledge of the vectorial field distribution of the holographic image, including SoP properties. On each map, the inset emphasizes the complex nature of all maps, and shows the color coding with phase encoded as hue and modulus as brightness.

**Supplementary Figure 10. Beam deflector with arbitrary polarization reconstruction.** (a) Structure configuration for the polarization generation of LP-45° (first row), LP-H (second row), RCP (third row), and EP (last row). (b) SEM images of the fabrication results. The red scale bar is 1μm. (c) Experimental results of the far-field intensity pattern scanning from -10° to 50°. All the deflectors are designed with the deflected angle at 30°. (d) Measured SoP of the deflected beam at 30°. The red and blue dots indicate the designed and measured polarization, respectively. Another diffraction order presenting identical intensity distribution at the opposite angle of -*θ_t_* is obtained with polarization of $\left| n_{-\theta} \right\rangle=a_{R}\left| L \right\rangle+a_{L}e^{i2\delta}\left| R \right\rangle$, which for simplicity is not presented in this paper.


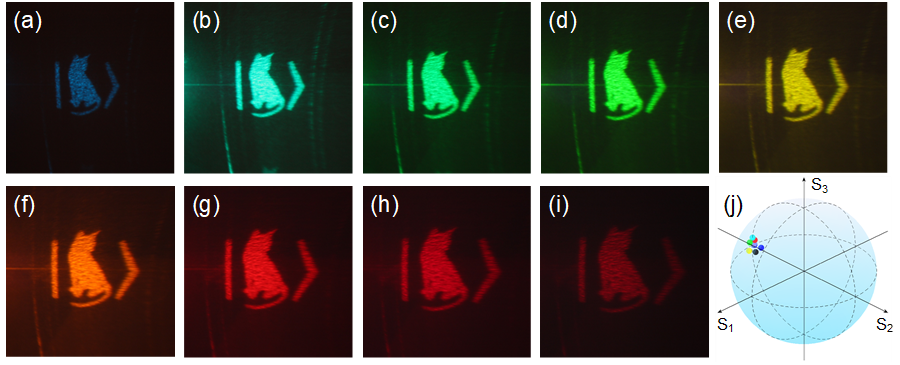


**Supplementary Figure 11. Measurement results in a wide bandwidth.** (a-i) The holographic images of “alive Schrödinger’s cat” representing the SoP of LP-45° with different incident wavelength from 475 nm to 675 nm. (j) The corresponding SoP of all the images on Poincaré sphere agrees well to the designed SoP of LP-45°, exhibiting broadband feature for output linear polarization.

**Supplementary Figure 12. Ghost image due to grating effect.** (a) Schematic of the grating effect due to a lattice constant of *d* between adjacent sub-pixels. The phase difference between adjacent sub-pixels $\Delta\varphi=k_{0}d\sin\theta_{t}$ is used to generate the holographic image. There is a series of *β_m_* (*m* = ±1, ±2…) that the phase difference between adjacent sub-pixels is equal to Δ*φ* + 2*mπ*, so that a series of ghost images will be induced. The angle *β_m_* satisfies the relationship of $\Delta\varphi+2m\pi=k_{0}d\sin\beta_{m}$. Combined with equation of $\Delta\varphi=k_{0}d\sin\theta_{t}$, the angle of the grating order can be derived as $\beta_{m}=\arcsin(\sin\theta_{t}+\frac{2m\pi}{k_{0}d})$. (b) Meta-hologram image designed with interested order *θ_t_* = 25° and lattice constant *d* = 8*p* = 2.4 μm. Ghost images at 10° and 42° are produced, which agree well to the theoretical results (*β*_-1_ = 9.94°, *β*_+1_ = 42.27°).


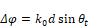

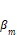

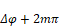

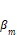

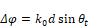

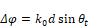

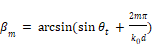


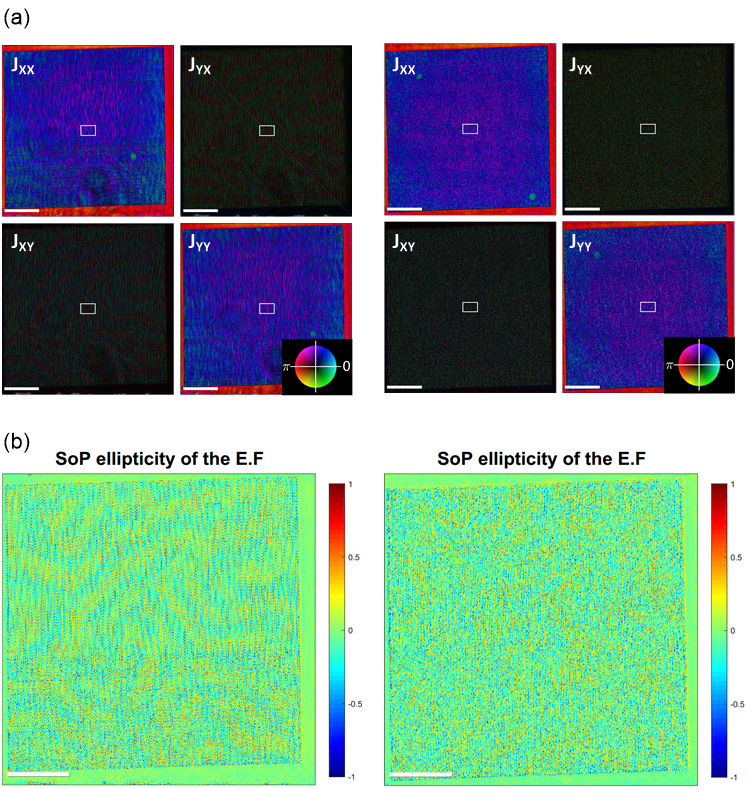


**Supplementary Figure 13. Measurement results by vectorial ptychography.** (a) Jones matrix maps of the meta-hologram. The enlarged images of the central rectangle area are shown in Fig. 4f and 4g. Scale bars are 50 µm. (b) Exit field over the entire metasurface. The colormap encodes the ellipticity value (1 = RCP, 0 = LP, -1 = LCP). Scale bars are 50 µm. (Left: uniformly distributed meta-hologram. Right: randomly distributed meta-hologram.)

**Supplementary Figure 14. Reconstructed far field pattern of the metasurface by vectorial ptychography.** (a) SoP orientation map (in degree), (b) SoP ellipticity value map (1 = RCP, 0 = LP, -1 = LCP) and (c) intensity values (arbitrary units) together with the corresponding SoP, when illuminated by a LP-H plane wave. Pixels corresponding to lower far-field intensities are displayed in black. (Left: uniformly distributed meta-hologram. Right: randomly distributed meta-hologram.)
